# Dual Role of φ29 DNA Polymerase Lys529 in Stabilisation of the DNA Priming-Terminus and the Terminal Protein-Priming Residue at the Polymerisation Site

**DOI:** 10.1371/journal.pone.0072765

**Published:** 2013-09-04

**Authors:** Alicia del Prado, José M. Lázaro, Laurentino Villar, Margarita Salas, Miguel de Vega

**Affiliations:** Instituto de Biología Molecular “Eladio Viñuela” (CSIC), Centro de Biología Molecular “Severo Ochoa” (CSIC-UAM), Universidad Autónoma, Cantoblanco, Madrid, Spain; Tulane University Health Sciences Center, United States of America

## Abstract

Resolution of the crystallographic structure of φ29 DNA polymerase binary and ternary complexes showed that residue Lys529, located at the C-terminus of the palm subdomain, establishes contacts with the 3′ terminal phosphodiester bond. In this paper, site-directed mutants at this Lys residue were used to analyse its functional importance for the synthetic activities of φ29 DNA polymerase, an enzyme that starts linear φ29 DNA replication using a terminal protein (TP) as primer. Our results show that single replacement of φ29 DNA polymerase residue Lys529 by Ala or Glu decreases the stabilisation of the primer-terminus at the polymerisation active site, impairing both the insertion of the incoming nucleotide when DNA and TP are used as primers and the translocation step required for the next incoming nucleotide incorporation. In addition, combination of the DNA polymerase mutants with a TP derivative at residue Glu233, neighbour to the priming residue Ser232, leads us to infer a direct contact between Lys529 and Glu233 for initiation of TP-DNA replication. Altogether, the results are compatible with a sequential binding of φ29 DNA polymerase residue Lys529 with TP and DNA during replication of TP-DNA.

## Introduction

**Figure 1:**
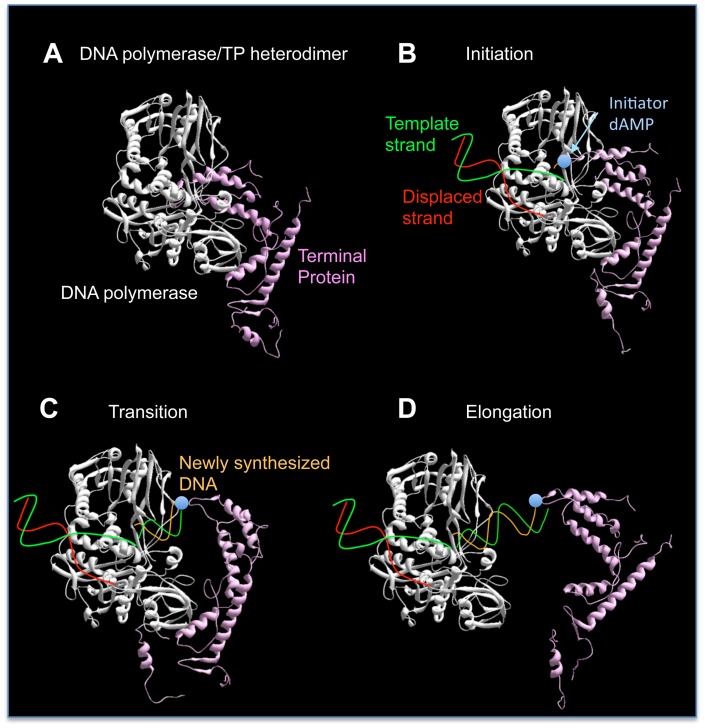
Schematic representation of the initial steps during φ29 TP-DNA replication. φ29 DNA polymerase is coloured in white, TP in pink, template strand in green, displaced strand in red, growing strand in light orange and the initiator dAMP as a blue circle. Crystallographic data corresponding to φ29 DNA polymerase/TP heterodimer are from Protein Data Bank (PDB) ID 2EX3 [35]. See main text for details.

Several prokaryotic and eukaryotic viruses, as well as linear plasmids from bacteria, fungi and higher plants, and even *Streptomyces* spp., have a TP protein (called Terminal Protein; TP) covalently linked to the 5′ DNA ends that, in several cases, has been shown to prime DNA synthesis from the very end of their linear genomes [1]–[3]. In these cases, a specific amino acid (Ser, Tyr or Thr) of the TP provides the priming OH group, and therefore the protein becomes covalently linked to the 5′-end of the growing DNA strand (parental TP). *In vitro* replication analyses, mainly performed with bacteriophage φ29, have laid the foundations for this so-called protein-priming replication mechanism. Briefly, the complex formed between the replicative DNA polymerase and a free TP molecule (Figure 1A) recognises the replication origins, placed at both ends of the genome and constituted by the parental TP and short inverted terminal repeats. Once bound at the replication origins, the DNA polymerase of the heterodimer catalyses the incorporation of a specific dNMP onto the OH group of the TP priming residue, in a reaction directed by an internal dNMP in the template strand (initiation reaction, see Figure 1B). The presence of repetitive sequences at the replication origins in these genomes allows further recovering of the terminal nucleotides by a backward movement of the initiation complex called sliding-back, described in bacteriophages φ29 [4], GA-1 [5], PRD1 [6] and Cp-1 [7], or jumping-back in adenovirus [8]. The DNA polymerase remains bound to the TP during the insertion of the first 9 nucleotides, a stage known as transition [9] (Figure 1C). Once the tenth nucleotide is added, the polymerase dissociates from the TP and the same DNA polymerase molecule fulfils TP-DNA replication *via* a strand displacement mechanism [2] (Figure 1D).

Bacteriophage φ29 DNA polymerase is the only enzyme fully responsible for viral DNA replication [10] due to its distinctive properties: extremely high processivity that allows it to replicate the entire genome from a single binding (and priming) event, without the assistance of processivity factors [11], and efficient coupling of DNA polymerisation to strand displacement that makes unnecessary the participation of DNA unwinding proteins [11]. In addition, φ29 DNA polymerase is the only member of the protein-primed subgroup of DNA polymerases whose structure has been solved [12]. It consists of a N-terminal exonuclease domain that contains the catalytic residues involved in proofreading of the misinserted nucleotides, and a C-terminal polymerisation domain having those residues responsible for DNA synthesis. The latter domain has two insertions called Terminal Protein Region 1 and 2 (TPR1 and TPR2), specifically present in the protein-primed DNA polymerase subgroup. These insertions, together with the universal palm, thumb and fingers subdomains, form an internal ring-like structure that encircles the upstream duplex product during DNA-primed elongation [12], [13] providing the enzyme with its inherent high processivity and strand-displacement capacity [14].

**Figure 2:**
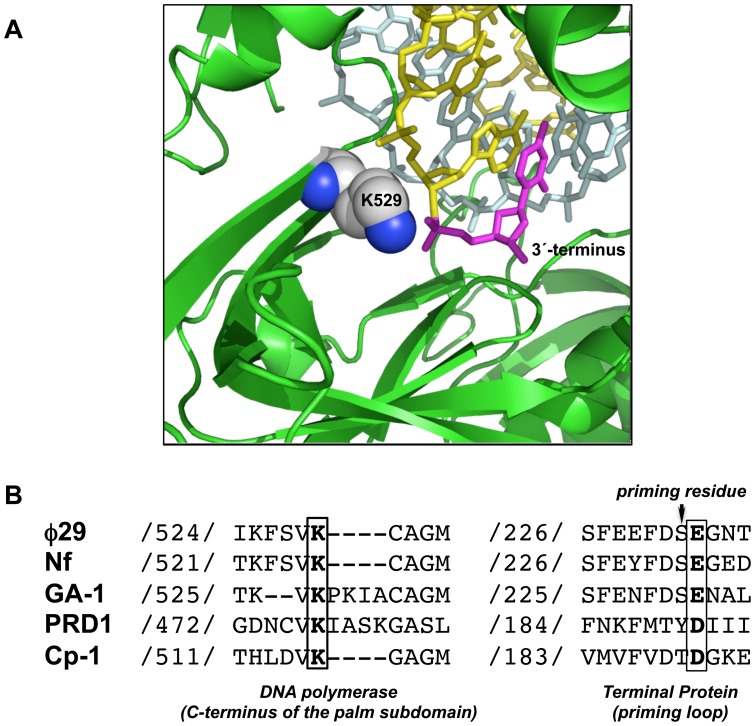
φ29 DNA polymerase residue Lys529. (A) Crystallographic data corresponding to φ29 DNA polymerase binary complex are from Protein Data Bank (PDB) ID 2PZS [13]. A detailed picture shows residue Lys529 (represented as spheres), placed at the C-terminus of the palm subdomain, interacting with the phosphates of the last phosphodiester bond. Primer and template strands are depicted as yellow and cyan sticks, respectively. The 3′ terminal nucleotide of the primer strand, placed at the priming site of the polymerase, is colored in magenta. (B) Amino acid sequence alignment of the C-terminal region of the palm subdomain and TP-priming loop of protein-primed DNA polymerases and TPs, respectively. DNA polymerases accession numbers are as follow: φ29 DNA polymerase (GenBank accession number CAA37450.1), PRD1 DNA polymerase (NCBI reference sequence NP040682.1), Nf DNA polymerase (GenBank accession number ACH57069.1), GA-1 DNA polymerase (GenBank accession number CAA65712.1) and Cp-1 DNA polymerase (GenBank accession number Q37989). Numbers indicate the position of the first aligned amino acid with respect to the N-terminus of the corresponding DNA polymerase. The conserved Lys residue is written in bold letter. TPs accession numbers are as follow: φ29 (UniProtKB accession number P03681), GA-1 (NCBI Reference sequence NP073686.1), Nf (GenBank accession number ACH57070.1), PRD1 (GenBank AAA32449.1) and Cp-1 (NCBI Reference Sequence NP044816.1). Numbers indicate the position of the first aligned amino acid with respect to the N-terminus of the respective TP. The conserved Glu/Asp residue is written in bold letter. The priming residue is indicated with an arrow.

Further resolution of the crystallographic structure of φ29 DNA polymerase binary and ternary complexes revealed a mechanism of translocation that seemed to be assisted by the coordinated movement of two conserved tyrosine residues into the nucleotide insertion site [13]. In addition, those structures showed that the polymerase makes contacts with the sugar-phosphate backbone of duplex DNA through a few direct interactions and multiple water-mediated hydrogen bonds [13]. Among them, residue Lys529, located at the C-terminus of the palm subdomain, establishes a water-mediated contact with one of the phosphate groups of the phosphodiester bond between the 3′ terminal and penultimate nucleotides (see Figure 2A). Sequence alignment of the DNA polymerases from phages that start replication by protein-priming as Nf, GA-1, PRD1 or Cp-1 shows a conservation of the corresponding Lys residue (see Figure 2B).

In this work we studied the role of φ29 DNA polymerase residue Lys529 by functional analyses of site directed mutants. The results allow us to suggest a role for this residue in the stabilisation of the primer-terminus at the polymerisation active site, required to guarantee proper nucleotide incorporation as well as the further translocation step. In addition, Lys529 contributes to the coordination between the polymerisation and 3′-5′ exonuclease activities. Biochemical assays performed by combining the DNA polymerase mutants with a TP mutant at residue Glu233, neighbour to the priming Ser232, lead us to infer a direct contact between both residues required to allow initiation of TP-DNA replication.

## Materials and Methods

### Nucleotides and DNAs

Unlabelled nucleotides were supplied by Amersham Pharmacia. [α-^32^P] dATP (3000 Ci/mmol) and [γ-^32^P]ATP (3000 Ci/mmol) were supplied by Perkin Elmer. Oligonucleotides were obtained from Invitrogen.

Oligonucleotides 15mer (5′-GATCACAGTGAGTAC), 30mer (5′-AACTGCCAAGAATAGTGTCAGTTCCAGACG), 5mer (5′-GATCA) and 4mer (5′-GATC) were purified electrophoretically on 8 M urea-20% polyacrylamide gels and 5′-labelled with [γ-^32^P]ATP and phage T4 polynucleotide kinase. Labelled oligonucleotides 15mer and 30mer were hybridized to oligonucleotide 21mer (5′-TCTATTGTACTCACTGTGATC) and 36mer (5′ TCTATTCGTCTGGAACTGACACTATTCTTGGCAGTT) respectively, in the presence of 0.2 M NaCl and 50 mM Tris-HCl, pH 7.5, resulting in primer/template structures.

### Site-directed mutagenesis of φ29 TP and DNA polymerase

TP and DNA polymerase mutants were obtained using the QuikChange site-directed mutagenesis kit provided by Stratagene, using as template plasmid pT7-3 that contains the viral gene 3 coding for the wild-type φ29 TP [15] and either plasmid pJLPM [16] (a derivative of pT7-4w2) containing the viral gene 2 that encodes the wild-type φ29 DNA polymerase or plasmid pT7-3 harboring the φ29 DNA polymerase exonuclease deficient mutant D12A/D66A [17]. The presence of the desired mutations, as well as the absence of additional ones was determined by sequencing the entire gene. TP and DNA polymerase mutants were expressed in *Escherichia coli* BL21(DE3) cells and further purified essentially as described for the wild-type TP [18] and wild-type DNA polymerase [16].

### Polymerase/3′-5′ exonuclease (pol/exo) coupled assay

The primer/template molecule 15mer/21mer (double-stranded DNA; dsDNA) contains a 6 nt 5′-protruding end and therefore can be used as substrate for the exonuclease activity (dsDNA) and also for DNA-dependent DNA polymerisation. The incubation mixture contained, in 12.5 µl, 50 mM Tris–HCl, pH 7.5, 10 mM MgCl_2_, 1 mM DTT, 4% (v/v) glycerol, 0.1 mg/ml BSA, 1.2 nM of 5′-labelled 15mer/21mer substrate, 30 nM of wild-type or mutant φ29 DNA polymerase and the indicated increasing concentrations of the four dNTPs. After incubation for 5 min at 25°C, the reaction was stopped by adding EDTA up to a final concentration of 10 mM. Samples were analysed by electrophoresis in 8 M urea-20% polyacrylamide gels and autoradiography. Polymerisation or 3′-5′ exonucleolysis is detected as an increase or decrease, respectively, in the size (15mer) of the 5′-labelled primer.

### Polymerisation assay of exonuclease deficient variants of φ29 DNA polymerase

The reaction was performed essentially as described for the Pol/Exo assay in the presence of 30 nM of the indicated exonuclease deficient variant of the φ29 DNA polymerase and the indicated concentrations of the four dNTPs. After incubation for 5 min at 25°C, the reaction was stopped by adding EDTA up to a final concentration of 10 mM. Samples were analysed by electrophoresis in 8 M urea-20% polyacrylamide gels and autoradiography.

### Measurement of the K_m_ for the incoming nucleotide

The incubation mixture contained, in a final volume of 12.5 µl, 50 mM Tris-HCl, pH 7.5, 1 mM DTT, 4% (v/v) glycerol, 0.1 mg/ml BSA, 10 mM MgCl_2_ and 1.2 nM of the primer/template molecule. Reaction times and enzyme concentration were adjusted for each polymerase to optimize product detection while ensuring that all reactions were conducted in the steady-state. Only those reactions that fell within the linear range of substrate utilization (<30% primer extension) were used for analysis. Samples were incubated for 15 sec at 30°C in the presence of increasing concentration of the incoming nucleotide, and quenched by adding 10 mM EDTA. Reactions were analysed by electrophoresis in 8 M urea−20% polyacrylamide gels and quantified using a Molecular Dynamics PhosphorImager. Formation of the extended product was plotted against dNTP concentration. Apparent value for Michaelis-Menten constant (*K_m_*) was obtained by least-squares nonlinear regression to a rectangular hyperbola using Kaleidagraph 3.6.4 software. Data are shown as Mean ± S.D. corresponding to four independent measurements.

### Hydrolysis of *p-*nitrophenol-TMP

The incubation mixture contained, in a volume of 300 µl, 50 mM Tris-HCl, pH 8.0, 150 mM NaCl, 1 mM DTT, 1 mM MnCl_2,_ 3 mM of *p*-nitrophenol-TMP (*p*NP-TMP) dissolved in 50 mM Tris-HCl, pH 8.0 and 150 mM NaCl, and 500 nM of either the wild-type or the indicated mutant φ29 DNA polymerase. Hydrolysis was studied by monitoring *p*-nitrophenol production at 420 nm with a Hitachi U-200 spectophotomer at 25°C, essentially as described [19]. Production of *p*-nitrophenol was plotted against time and adjusted to a rectangular hyperbola by least-squares non-linear regression, using Kaleidagraph 3.6.4. software. Linearity in the production of *p*-nitrophenol was obtained in the time range 5–100 s. Slopes obtained by linear regression adjustments of those points allowed to calculate the activity for the hydrolysis of the phosphoester bond (s^−1^).

### 3′-5′ Exonuclease assay

The incubation mixture contained, in a final volume of 12.5 µl, 50 mM Tris-HCl, pH 7.5, 1 mM DTT, 4% (v/v) glycerol, 0.1 mg/ml BSA, 10 mM MgCl_2_ and 12 nM of either the wild-type or the indicated φ29 DNA polymerase variant. As ssDNA substrate, 1.2 nM of the 5′-labelled 15mer, 14.5 nM of the 5mer oligonucleotide or 18 nM of the 4mer oligonucleotide was used. Analysis of the exonuclease activity on dsDNA was performed using as substrate 1.2 nM of the hybrid molecule 15mer/21mer. Samples were incubated at 25°C for the indicated times and quenched by adding EDTA up to a final concentration of 10 mM. Reactions were analysed by electrophoresis in 8 M urea−20% polyacrylamide gels and densitometry of the autoradiograph. Total degradation was obtained by calculating the number of catalytic events giving rise to each degradation product. From these data, the catalytic efficiency of each mutant derivative was calculated relative to the wild-type φ29 DNA polymerase.

### Exonuclease activity under single-turnover conditions

The incubation mixture contained, in 12.5 µl, 50 mM Tris–HCl, pH 7.5, 1 mM DTT, 4% (v/v) glycerol, 0.1 mg/ml BSA, 12 nM of wild-type or the indicated mutant φ29 DNA polymerase and 0.36 nM of the 5′-labelled 30mer/36mer molecule. After incubation for 15 min at 4°C to allow the formation of a complex between the DNA polymerase and the DNA substrate for exonucleolysis, the reaction was started by addition of 10 mM MgCl_2_ and 0.36 µM of the unlabelled 30mer/36mer as challenger DNA. Samples were incubated for the indicated times at 25°C and the reaction was stopped by adding EDTA up to a final concentration of 10 mM. Samples were analysed by electrophoresis in 8 M urea-20% polyacrylamide gels and autoradiography.

### DNA gel retardation assay

The interaction of either the wild-type or the φ29 DNA polymerase mutants with the primer–template structure was assayed using as substrate the 5′-labelled 15mer/21mer. The incubation mixture contained, in a final volume of 20 µl, 12 mM Tris-HCl, pH 7.5, 1 mM EDTA, 20 mM ammonium sulphate, 0.1 mg/ml BSA, 10 mM MgCl_2_, 0.7 nM of the 15mer/21mer DNA molecule and the indicated amount of wild-type or the indicated mutant φ29 DNA polymerase. After incubation for 5 min at 4°C, the samples were subjected to electrophoresis in precooled 4% (w/v) polyacrylamide gels (acrylamide/bis-acrylamide 80∶1, w/w) containing 12 mM Tris-acetate, pH 7.5 and 1 mM EDTA, and run at 4°C in the same buffer at 8 V/cm [20]. After autoradiography, φ29 DNA polymerase/DNA stable interaction was detected as a shift (retardation) in the migrating position of the labelled DNA and quantified by densitometry of the autoradiograms corresponding to different experiments.

### Protein-primed initiation assay (TP-dAMP formation)

The ability to carry out the initiation step during TP-DNA replication was analysed essentially as described [21]. The incubation mixture contained, in 25 µl, 50 mM Tris–HCl, pH 7.5, 10 mM MgCl_2_, 20 mM ammonium sulphate, 1 mM DTT, 4% (v/v) glycerol, 1.6 nM of φ29 TP-DNA as template, 0.1 mg/ml BSA, 0.2 µM dATP (1µCi [α-^32^P] dATP), 13 nM of either wild-type or mutant TP and 13 nM of either wild-type or the indicated mutant DNA polymerase. Samples were incubated for 4 min at 30°C. Reactions were stopped by adding 10 mM EDTA-0.1% SDS, and the samples were filtered through Sephadex G-50 spin columns and further analysed by SDS–12% polyacrylamide gels. Quantitation was done by densitometric analysis of the labelled band corresponding to the TP-dAMP complex detected by autoradiography.

### TP-DNA replication assay

The replication assay was performed essentially as described [21]. The incubation mixture contained, in 25 µl, 50 mM Tris-HCl, pH 7.5, 10 mM MgCl_2_, 20 mM ammonium sulphate, 1 mM DTT, 4% (v/v) glycerol, 0.1 mg/ml BSA, 20 µM each dNTP and [α-^32^P]dATP (1 µCi), 13 nM of either wild-type or mutant TP, 13 nM of either wild-type or mutant DNA polymerase, and 1.6 nM of φ29 TP-DNA. After incubation for 10 minutes at 30°C, the reaction was stopped by adding 10 mM EDTA−0.1% SDS, and the samples were filtered through Sephadex G-50 spin columns. Quantitation of the DNA synthesized *in vitro* was carried out from the amount of radioactivity (Cerenkov radiation) corresponding to the excluded volume. The labelled DNA was denatured by treatment with 0.7 M NaOH and subjected to electrophoresis in alkaline 0.7% agarose gels, as described [22]. After electrophoresis, the position of unit-length φ29 DNA (19285 nucleotides) was detected by ethidium bromide staining, and then the gels were dried and autoradiographed.

### Transition assay

The assay was performed essentially as described for the TP-DNA replication assay. For the analysis of the transition products, 13 nM of either wild-type or mutant TP, 13 nM of wild-type or the indicated mutant DNA polymerase and 1.6 nM of TP-DNA were incubated in the presence of 5 µM dATP, dGTP and dTTP for 5 min at 30°C. The reaction was stopped by adding 10 mM EDTA-0.1% SDS, and the samples were filtered through Sephadex G-50 spin columns. The samples were analysed by electrophoresis in SDS-12% polyacrylamide gels (360×280×0.5 mm) to obtain enough resolution to distinguish the TP bound to the first elongation products.

### Analysis of the interaction between TP and DNA polymerase mutants by glycerol-gradient ultracentrifugation

The assay was performed essentially as described [21]. The incubation mixture contained, in 150 µl, 50 mM Tris–HCl, pH 7.5, 1 mM DTT, 0.1 mg/ml BSA, 20 mM ammonium sulphate, 0.6 µM of either wild-type or mutant DNA polymerase and 0.6 µM of wild-type TP. After incubation for 30 min at 4°C, samples were loaded on top of a continuous 15–30% (v/v) glycerol gradient (4 ml) in the presence of 50 mM Tris-HCl, pH 7.5, 20 mM ammonium sulphate, 0.2 M NaCl, 1 mM EDTA and 7 mM β-mercaptoethanol, and centrifuged at 4°C for 24 h at 58000 rpm in a Beckman TST 60.4 rotor. Gradients were fractionated and subjected to SDS-12% polyacrylamide gel electrophoresis. The proteins in the gel were stained with SYPRO to identify the peaks corresponding to the TP/DNA polymerase heterodimer (97 kDa) and the free monomers of TP (31 kDa) and DNA polymerase (66 kDa).

## Results and Discussion

### Site-directed mutagenesis of φ29 DNA polymerase residue Lys529

To analyse the role of the above mentioned Lys residue in the stabilisation of the primer-terminus at the polymerisation active site, as well as in the coordination of the polymerisation and 3′-5′ exonuclease activities, the φ29 DNA polymerase residue Lys529 was changed into Ala (mutant K529A) to remove the positive charge, and into Glu to introduce a negatively charged amino acid. The mutant derivatives were overexpressed and purified as described in Materials and Methods, and their catalytic efficiencies analysed by *in vitro* biochemical assays.

### Mutations at φ29 DNA polymerase residue Lys529 affect the proper balance between polymerisation and 3′-5′ exonucleolysis of the DNA polymerase

Most DNA-dependent DNA polymerases are endowed with at least two catalytic activities, DNA polymerisation and 3′-5′ exonucleolysis, governed by catalytic sites present in two structurally distant domains [12], [23]–[26]. Despite this physical separation, these two opposite activities must act in concert to achieve a productive and accurate replication reaction. The decision to extend *versus* to reduce the primer length finally depends on the relative rate of each catalytic (polymerisation *vs* exonucleolysis) reaction. However, assuming a relatively constant temperature and ionic strength, and no significant variations of the dNTP pool under *in vivo* conditions, the main factor controlling this equilibrium is the relative stability of the primer-terminus (the common substrate) at both active sites [27]–[34].

**Figure 3:**
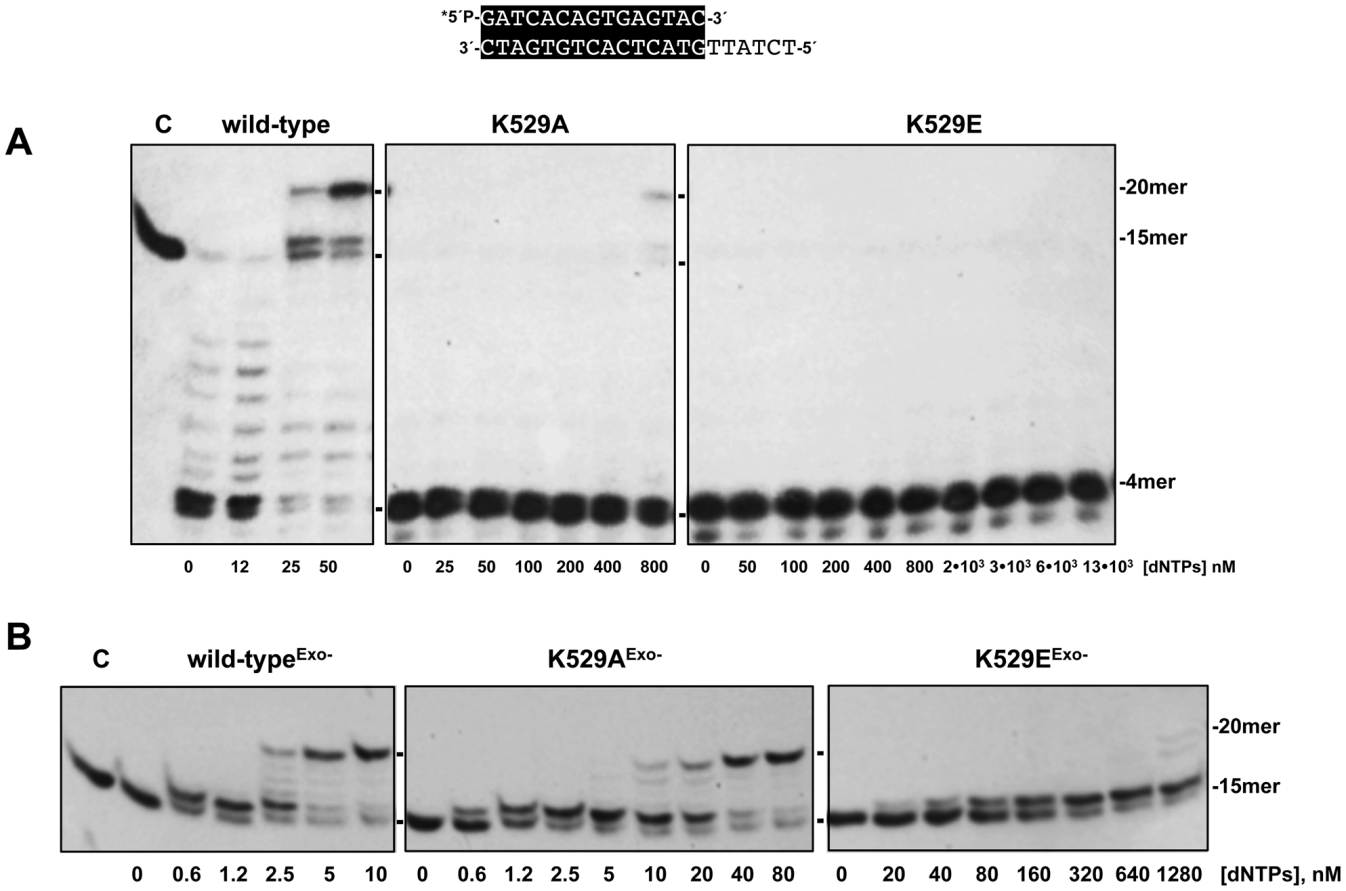
Mutations at φ29 DNA polymerase residue Lys529 affect the equilibrium between the polymerisation and the 3′-5′ exonuclease activities. (A) DNA polymerase/exonuclease coupled assay. The assay was performed as described in Materials and Methods using the 5′-labelled primer/template molecule 15mer/21mer depicted on top of the figure and the indicated concentration of dNTPs. Asterisk indicates the ^32^P 5′-labelled end of the primer strand. Polymerisation or 3′-5′ exonucleolysis is detected as an increase or decrease, respectively, in the size (15mer) of the 5′-labelled primer. (B) DNA polymerisation catalysed by wild-type^Exo−^ (D12A/D66A), K529A^Exo−^ (K529A/D12A/D66A) and K529E^Exo−^ (K529E/D12A/D66A) DNA polymerases. The assay was performed as described in (A), using as substrate he 5′-labelled 15mer/21mer molecule and the indicated concentration of dNTPs. C: control DNA.

To evaluate how the mutations introduced affected the dynamic equilibrium between the 3′-5′ exonuclease and polymerisation activities of the DNA polymerase, we studied the functional coupling between synthesis and degradation on a primer/template hybrid molecule (15mer/21mer) as a function of dNTP concentration. Without nucleotides, the only bands detected correspond to primer degradation products by the 3′-5′ exonuclease activity. As the concentration of the unlabelled dNTPs provided increases, the exonuclease activity is progressively competed by the 5′–3′ polymerisation one. Net dNTPs incorporation is observed as an increase in the size of the labelled primer, allowing to define the dNTP concentration needed to obtain an efficient elongation for each mutant derivative (Pol/Exo ratio). As shown in Figure 3A and Table 1, whereas the wild-type enzyme required 25–50 nM dNTPs to compete out its exonuclease activity, mutant K529A needed a concentration >32-fold higher (800 nM) to obtain some primer elongation. Mutant K529E did not render any elongation band even at the highest dNTPs concentration assayed (13 µM). It is noteworthy the strong 3′-5′ exonuclease activity exhibited by both mutants on the duplex DNA (compare lanes without nucleotides in Figure 3A).

**Table 1:** Enzymatic activities of wild-type and mutant φ29 DNA polymerases.

		φ29 DNA polymerase^a^		
Activity assay	Substrate	wild-type	K529A	K529E
Pol/Exo balance	primer/template^b^, dNTPs	25/50	>800	>13×10^3^
Polymerisation	primer/template^b^, dNTPs	2.5	20	>1280
3′-5′ exonuclease^c^	primer/template^b^	100	410±110	380±90
	ssDNA (15mer)	100	290±70	230±70
	ssDNA (5mer)	100	160±40	190±30
	ssDNA (4mer)	100	90±10	90±20
Km^d^	primer/template^b^, dATP	11.1±2.6	14.7±4.4	171±83

a Numbers indicate the dNTP concentration (in nM) required to efficiently elongate the 15mer primer until the 20mer position.

b 15mer/21mer hybrid molecule.

c Data represent the percent activity with respect to the wild-type DNA polymerase (100%) and the standard deviation obtained from at least three independent experiments.

d Km stands for Michaelis Mentent constant for the incoming nucleotide (in nM). Data are shown as Mean ± S.D. corresponding to four independent measurements.

When DNA is used as primer, any strong impairment in nucleotide incorporation, such as a mutation at the polymerisation active site, would unbalance the above mentioned equilibrium towards the exonucleolytic degradation of the primer, precluding a specific analysis of the synthetic features of the enzyme. Therefore, φ29 DNA polymerase mutants K529A and K529E were engineered to include the double mutation D12A/D66A [17] at two catalytic residues of the exonuclease active site to eliminate specifically their proofreading activity (mutants K529A^Exo−^ and K529E^Exo−^). As shown in Figure 3B (see also Table 1), despite the nearly wild-type proficiency displayed by mutant K529A^Exo−^ in the incorporation of the first nucleotide, even at the lowest dose of nucleotide analysed (K_m_  = 14.7±4.4 nM *versus* the wild-type K_m_ = 11.1±2.6 nM), it required a dNTP concentration 8-fold higher than the wild-type^Exo−^ enzyme to yield the elongated 20mer product. In contrast, primer elongation with mutant K529E^Exo−^ was severely impaired and mostly limited to the addition of only one nucleotide (K_m_  = 171±83 nM), even at the highest dNTP concentration assayed. The affinity for primer/template DNA molecules of wild-type and φ29 DNA polymerase mutants was directly studied using gel retardation assays, as described under Materials and Methods. Under these conditions, the wild-type φ29 DNA polymerase produces a single retardation band using a labelled hybrid 15mer/21mer molecule (see Figure S1) that has been interpreted as an enzyme-DNA complex competent for polymerization [29]. As also shown in Figure S1, φ29 DNA polymerase mutants showed a 2-fold reduced binding efficiency. Therefore, the deficient nucleotide incorporation exhibited by the mutant DNA polymerases would correlate with their decreased capacity to stabilise a primer-terminus at the polymerisation active site, in agreement with a primer-terminus binding role for Lys529.

Once the phosphoryl transfer reaction has taken place, the newly incorporated nucleotide has to move back from the insertion site to the priming site to allow the next incoming nucleotide incorporation. This translocation step makes possible processive movement of the polymerase along the template DNA, a critical characteristic of the nucleotide addition cycle of replicative polymerases. Structural analysis of the pre- and post-translocated states of φ29 DNA polymerase revealed a mechanism of translocation relying on the coordinated movement of two conserved tyrosine residues, concomitant with the opening of the fingers that takes place immediately after the insertion of the incoming nucleotide. Thus, such tyrosines enter the insertion site occupied by the recently inserted nucleotide, promoting the backward movement of the primer-terminus to the post-translocation priming site [13]. Therefore, based on those crystallographic structures, it can be predicted that once the incoming nucleotide has been inserted, the initial interaction between φ29 DNA polymerase Lys529 and the 3′ terminal phosphodiester bond must be broken during translocation to allow the side chain of the amino acid to interact with the newly formed phosphodiester bond at the postranslocation state. This hypothesis agrees with the impairment displayed by mutants to synthesize products longer than one nucleotide, a result that stresses the importance of Lys529, not only during nucleotide insertion, but also in the further movement of the growing chain from the insertion site to the priming site to allow the next nucleotide addition, emphasizing the significance of the stabilisation of the primer-terminus at this critical stage of the polymerisation cycle.

### Mutations at residue Lys529 of φ29 DNA polymerase favor primer-terminus transference to the 3′-5′ exonuclease active site

**Figure 4:**
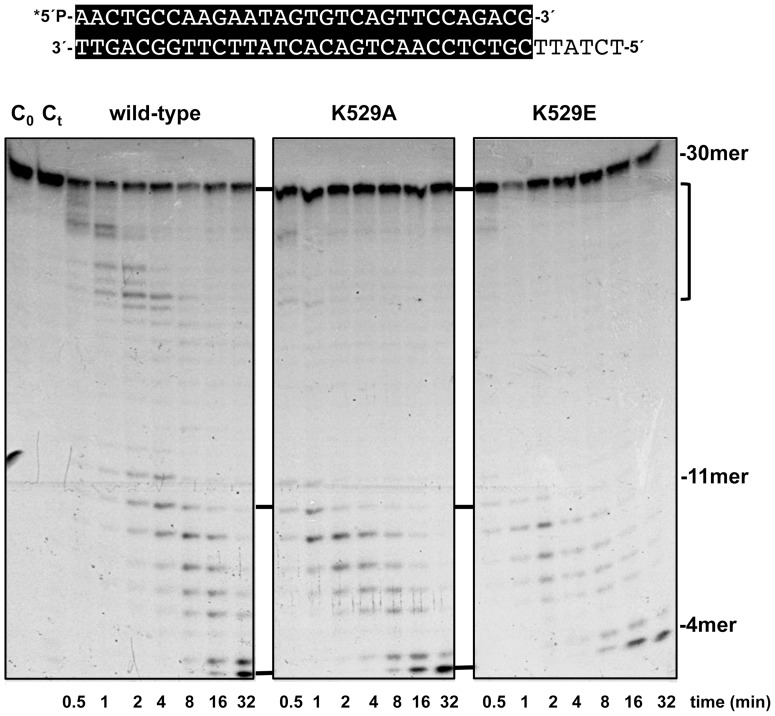
3′-5′ exonuclease activity of mutants K529A and K529E under single binding event conditions. The assay was carried out as described in Materials and Methods using as substrate the 5′ P-labelled 30mer/36mer primer/template depicted on top of the figure. Asterisk indicates the ^32^P 5′-labelled end of the primer strand. The exonucleolytic reaction was initiated by addition of the metal activator and a 100-fold excess of nonlabelled substrate as challenger DNA. After incubation at 25°C for the times indicated, the reactions were stopped by addition of EDTA to 10 mM and analysed by electrophoresis in 8 M urea-20% polyacrylamide gels and autoradiography. C_o_: control DNA. C_t_: preincubation of DNA polymerase with a 100-fold excess of the nonlabelled substrate as challenger DNA.

As mentioned above, the mutant derivatives exhibited an enhanced exonucleolytic activity on the primer/template DNA substrate. Time-course experiments on the 15mer/21mer molecules showed that the 3′-5′ exonuclease activity of both mutants was 4-fold higher than that of the wild-type enzyme (see Table 1 and Figure S2A). To ascertain the wild-type folding of the catalytic site that could be responsible for the higher exonucleolytic proficiency of the mutants, their ability to hydrolyze the 5′-*p*-nitrophenyl ester of thymidine 5′-monophosphate (pNP-TMP) was analysed as described [19] (see Figure S3). This is a minimal substrate for the exonuclease activity whose binding for further hydrolysis relies solely on those ligands responsible for the stabilisation of the 3′ terminal nucleotide of a ssDNA during exonucleolysis. The rate of hydrolysis of pNP-TMP by the mutant enzymes, determined spectrophotometrically by continuous monitoring of the *p*-nitrophenol produced, was calculated to be 0.32 s^−1^, close to that of the wild-type enzyme (0.25 s^−1^). Once established a wild-type folding of the exonuclease active site of the mutant derivatives, we analysed the degradation of a 5′-labelled 30mer/36mer primer/template molecule under single-turnover conditions in the presence of a 100-fold excess of non-labelled substrate (challenger DNA). Under these conditions, all DNA polymerase molecules not bound to (or dissociated from) the 5′-labelled substrate molecules will be trapped by the challenger DNA, allowing the analysis of the exonucleolytic degradation from a single binding event. As it can be seen in Figure 4 (lane C_t_) the amount of challenger DNA used was enough to trap all the DNA polymerase molecules since when it was simultaneously added to the reaction mixture no degradation of the labelled hybrid was observed. When the wild-type φ29 DNA polymerase was preincubated with the labelled substrate before the addition of metal and challenger DNA, the enzyme rendered several intermediate degradation products (19–25mer) at the lowest reaction times (Figure 4). Such a pattern is interpreted as pauses during exonucleolytic digestion of the DNA that occur without dissociation of the enzyme, as those intermediate products are progressively shortened at longer reaction times. Mutant K529A, and at a higher extent K529E, did not give those intermediate stops, resulting in a higher degradation rate. These results would indicate that residue Lys529 could act as a barrier to prevent overdegradation of the substrates, regulating the exonuclease activity of the enzyme. The results agree with the recent proposal of a primer partitioning model between both active sites in φ29 DNA polymerase that would be accomplished by a passive diffusion of the frayed primer-terminus from the polymerisation to the exonuclease site [30]. Such a model requires breaking of specific interactions responsible for the stabilisation of the primer at the polymerisation active site, creating new ones with residues specialized in binding the ssDNA at the exonuclease active site. Therefore, the poor stabilisation of the primer-terminus in the mutant derivatives would enhance its diffusion to the exonuclease active site.

When time-course experiments were performed on ssDNA (15mer molecule), the exonucleolytic activity of both mutant enzymes with respect to the wild-type polymerase was lower than when dsDNA was used as substrate (see Table 1 and Figure S2B), although it remained 2.9- (K529A) and 2.3-fold (K529E) higher, respectively, than that of the wild-type enzyme (see Table 1). The results could indicate that the ssDNA follows the same pathway as the primer-terminus towards the exonuclease active site, Lys529 making contacts with this substrate to limit its degradation. Considering the location of residue Lys529 at the palm subdomain of the polymerisation domain, degradation of short substrates should be insensitive to the mutations introduced at the lysine residue, as the enzyme-DNA interaction should rely only on specific residues located close to the exonuclease active site. In this sense, and as it can be observed in Table 1 (see also Figure S4), whereas mutant polymerases were still 2-fold more active than the wild-type enzyme on the 5mer substrate, their relative activity decreased to wild-type levels on the shortest substrate assayed (4mer). Based on the crystallographic structure of the φ29 DNA polymerase with an oligo (dT)_5_ bound at its exonuclease active site, Lys529 appears to contact with the 5′ nucleotide [12] (see Figure S5), explaining the higher exonuclease activity displayed by mutant polymerases on the 5mer substrate. Based on these structures, the 5′ nucleotide of a 4mer substrate could not establish contacts with Lys529, accounting for the wild-type exonuclease activity exhibited by mutant derivatives on this substrate.

### Role of φ29 DNA polymerase residue Lys529 during the first steps of TP-DNA replication

Crystallographic resolution of φ29 DNA polymerase/TP complex showed the extended structure of TP complementary to the DNA polymerase surface [35]. TP folds into a N-terminal domain that possesses sequence-independent DNA-binding capacity and is responsible for TP nucleoid association [36], an intermediate domain whose interaction with the DNA polymerase subdomain TPR-1 is the one mainly responsible for the specificity between both proteins and for the stability of the heterodimer [37], [38], and a C-terminal priming domain. The latter mimics duplex product DNA in its electrostatic profile and binding site in the polymerase, making extensive interactions with the TPR2 and thumb subdomains of the polymerase [35], [38]. It harbors the Ser232 residue placed closest to the active site of the DNA polymerase. Along with its role as primer of the initiation reaction, the TP priming domain also dictates the internal 3′ nucleotide used as template during initiation of replication [39].

**Figure 5:**
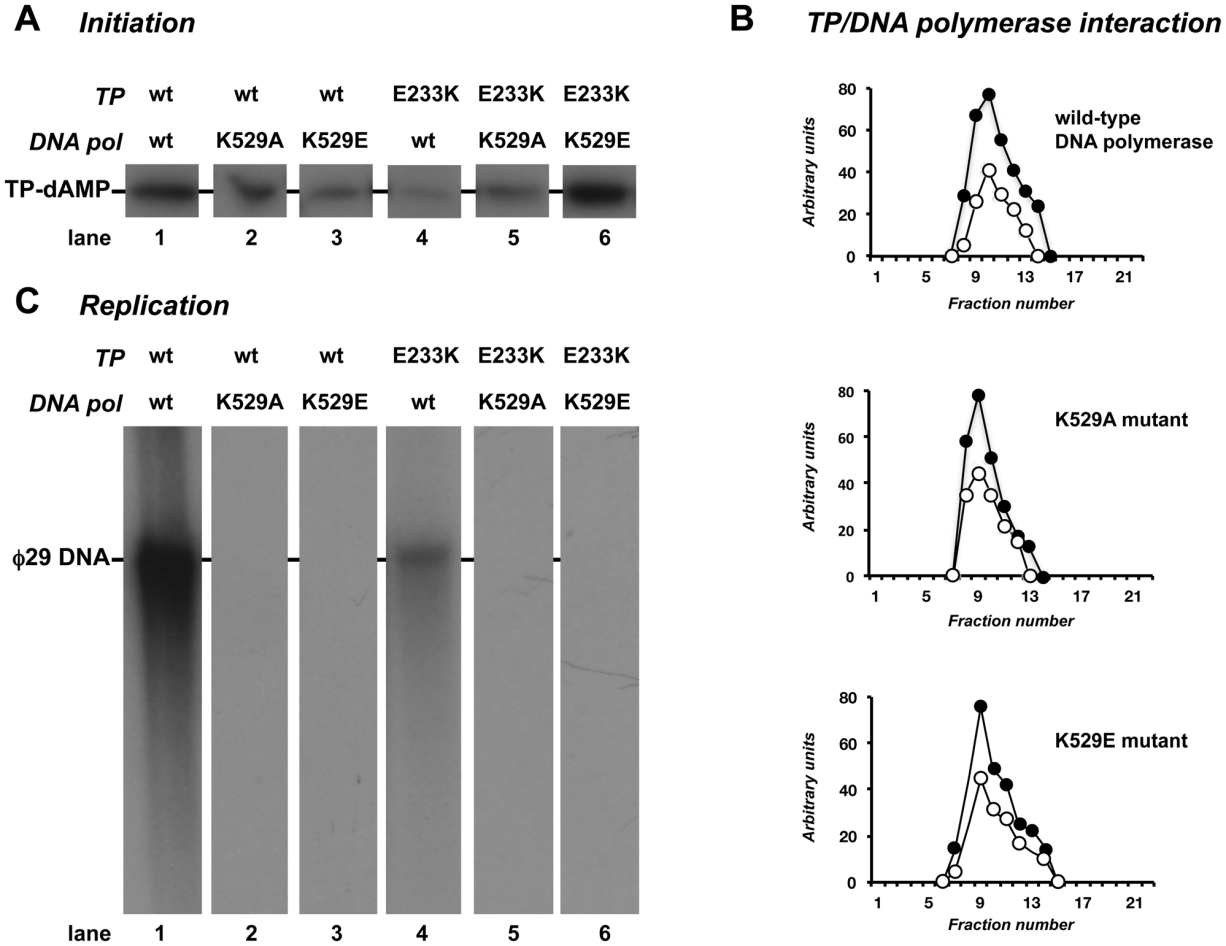
φ29 TP-DNA replication by mutants at residue Lys529 of φ29 DNA polymerase. (A) *In vitro* protein-primed initiation. The initiation assay was performed as described in Materials and Methods, in the presence of 13 nM of either wild-type or the indicated mutant TP, 13 nM of either wild-type or the indicated mutant DNA polymerase and 1.6 nM of φ29 TP-DNA. After 4 min incubation at 30°C, the reactions were stopped, processed and analysed by SDS-12% polyacrylamide gels and autoradiography. The position of the TP-dAMP complex is indicated. (B) Analysis of TP/DNA polymerase interaction by glycerol gradient ultracentrifugation. The assay was carried out as described under Materials and Methods, pre-incubating 0.6 µM of wild-type TP with 0.6 µM of either the wild-type or the indicated mutant DNA polymerase. After incubation for 30 min at 4°C, samples were loaded on top of a continuous 15–30% glycerol gradient in the presence of 0.2 M NaCl. After centrifugation, the collected fractions were subjected to SDS-12% polyacrylamide gel electrophoresis and further stained with SYPRO. Densitometric quantification, expressed in arbitrary units, of both DNA polymerase (full circles) and TP (open circles) is represented. (C) TP-DNA replication. The assay was carried out as described in Materials and Methods in the presence of 13 nM of wild-type or the indicated mutant DNA polymerase, and 13 nM of wild-type or mutant TP. After incubation for 10 min at 30°C, relative activity values were calculated (see Table 1), and the length of the synthesized DNA was analysed by alkaline agarose gel electrophoresis. The migration position of unit length φ29 DNA is indicated.

The absence of an ordered conformation of the TP-priming loop in the crystal structures of the φ29 DNA polymerase/TP complex precludes the analysis of potential interactions of this TP region with residues of the polymerase. However, if the OH group of the priming Ser232 residue is placed in the priming position of the polymerase, analogously to the 3′ hydroxyl of the DNA in the binary complexes, φ29 DNA polymerase residue Lys529 would contact TP residue Glu233 (conserved among the TPs of related phages; see Figure 2B) that would mimic the last phosphodiester bond of the 3′ terminus of the DNA. To test this possibility, the ability of mutant polymerases to form the TP-dAMP complex (initiation reaction) was evaluated using as template φ29 TP-DNA. As shown in lanes 2 and 3 of Figure 5A (see also Table 2), mutant polymerases were 2- (K529A) and 4-fold (K529E) less efficient in the formation of the TP-dAMP complex than the wild-type enzyme (lane 1).

**Table 2:** TP-primed activities of wild-type and mutant φ29 DNA polymerases.

		Activity assayed^a^	
DNA polymerase	Terminal protein	TP-dAMP formation	TP-DNA replication
wild-type	wild-type	100	100
K529A	wild-type	56±20	0.6±0.3
K529E	wild-type	27±9	0.2±0.1
wild-type	E233K	24±6	14±5
K529A	E233K	41±12	1±0.1
K529E	E233K	123±36	0.4±0.2

a Data represent the percent activity of mutant heterodimer with respect to the wild-type one (100%) and the standard deviation obtained from at least three independent experiments.

The diminished TP-dAMP complex formation displayed by the mutant enzymes is not due to a lack of interaction with the TP as both formed a stable heterodimer with the TP (directly analysed by glycerol gradient ultracentrifugation, see Figure 5B). Instead, it could be due to either the absence of the specific contact with TP residue Glu233 (in mutant K529A) or to an electrostatic repulsion (in mutant K529E). To examine this possibility, the φ29 TP residue Glu233 was changed to Lys (mutant E233K) to introduce a positively charged amino acid that should restore the interaction with the DNA polymerase mutant K529E (see Materials and Methods). As shown in Figure 5A (see also Table 2), TP mutant E233K was impaired in the formation of the TP-dAMP complex with both, the wild-type and mutant K529A DNA polymerase (lanes 4 and 5, respectively). In contrast, the heterodimer E233K (TP)/K529E (DNA polymerase) exhibited a wild-type initiation reaction (lane 6), strongly suggesting that the DNA polymerase residue Lys529 interacts with the Glu233 of the TP during the first step of TP-DNA replication. Such an interaction would guarantee the proper stabilisation/orientation of the priming residue Ser232 at the polymerisation active site.

### φ29 DNA polymerase mutants are unable to switch from protein-priming to DNA priming

Once catalysed the formation of the TP-dAMP initiation product, the same DNA polymerase molecule elongates it *via* strand displacement to produce full-length φ29 DNA (Figure 5C, lane 1). Mutations at Lys529 residue impaired the DNA polymerase to perform TP-DNA replication, regardless the initiation levels exhibited with both the wild-type (lanes 2 and 3) and mutant TPs (lanes 5 and 6). In contrast, the detectable replication capability of the heterodimer formed by the TP mutant E233K and the wild-type DNA polymerase (lane 4) was consistent with the TP-dAMP formation level (see Table 2). This last result suggests that once TP-dAMP is formed, TP Glu233 residue does not play any further role in replication.

**Figure 6:**
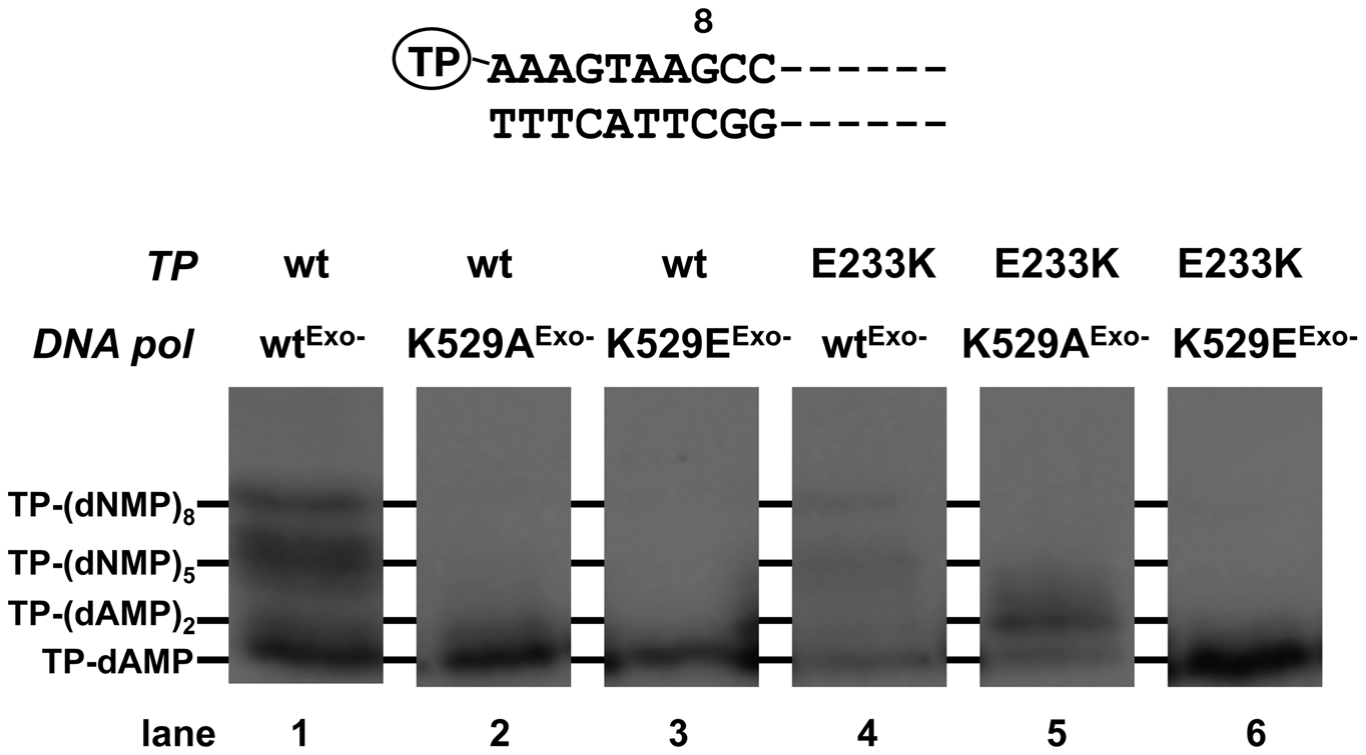
Analysis of the transition products of φ29 DNA replication. The assay was performed as described in Materials and Methods in the presence of 13 nM of the indicated DNA polymerase, 13 nM of either wild-type or the indicated mutant TP and 5 µM of each dATP, dGTP and dTTP. After incubation for 5 min at 30°C, the different transition products were detected and analysed by high resolution SDS-12% PAGE. The first 10 nucleotides of the left end of φ29 genome are depicted on top of the figure. Intermediate products yielded during the first replication steps of φ29 TP-DNA are indicated.

After the initiation step has taken place, the φ29 DNA polymerase/TP heterodimer remains as a complex. Once the 10th nucleotide is incorporated, the interaction between the two proteins is released to allow the same DNA polymerase molecule to continue replication [9]. This stage between the TP-primed and DNA-primed modes is known as transition. To analyse the transition step, the short synthesis products obtained in the presence of three dNTPs (without dCTP) were analysed by high-resolution gel electrophoresis. To better detect the amount of non-elongated initiation complexes and partially elongated products, all the DNA polymerases assayed were exonuclease deficient (see above), to prevent the degradation of the replication intermediates. As shown in lane 1 of Figure 6, the wild-type heterodimer gives rise to the initiation products TP-(dAMP)_1**–**2_, the intermediate transition molecules TP-(dNMP)_4**–**6_, described as molecules aborted during the transition from the initiation mode to the elongation one [9], and the TP-(dNMP)_8_ product that corresponds to the truncated elongation that started from the left origin of the TP-DNA. Despite the low initiation activity observed with the heterodimer formed by TP mutant E233K and wild-type DNA polymerase (lane 4), the ratio between the elongated products [TP-(dNMP)_4**–**8_] and the initiation complexes [TP-(dAMP)_1**–**2_] was similar to that obtained with the wild-type heterodimer, agreeing with the role of TP residue Glu233 restricted to the initiation step. In contrast, mutations at Lys529 hindered the DNA polymerase to progress further after the formation of the initiation products TP-(dAMP)_1**–**2_, regardless of their initiation level, either with the wild-type TP (lanes 2 and 3) or with the E233K mutant (lanes 5 and 6). It has been described that once DNA polymerase catalyses the formation of the TP-dAMP initiation product, a reaction directed by the second T at the 3′ end (3′-TTT) [4], it translocates one position backward to recover the template information corresponding to the first T, the so-called sliding-back mechanism. This step would be analogous to the translocation of the 3′ terminus of a growing DNA strand from the nucleotide insertion site to the priming site (described above). In this sense, and as it occurred with a DNA substrate, once the mutant polymerases accomplished the initiation reaction, they stalled after the formation of the initiation products. The impairment to proceed further would stress the importance of residue Lys529 in the backward movement of the φ29 DNA polymerase priming substrates, DNA and TP.

In this paper we have shown, by means of site directed mutants, the importance of φ29 DNA polymerase residue Lys529 in guaranteeing stabilization of the primer-terminus at the polymerisation active site and, as a consequence, in the insertion of the incoming nucleotide. In addition, the contact between residue Lys529 and the 3′ terminus is critical in the translocation step required for the next incoming nucleotide incorporation. Furthermore, although the structure of the TP loop that contains the priming Ser232 has not been solved, here we have inferred that TP residue Glu233, conserved in the TP of φ29-like phages, could be mimicking the last phosphodiester bond of a conventional DNA 3′ terminus. Combinations of the DNA polymerase mutants at residue Lys529 with a TP derivative at residue Glu233, neighbour to the priming Ser232, lead us to propose a direct contact between both residues required for the initiation of TP-DNA replication and further translocation to allow complete genome replication. Altogether, the results are compatible with a sequential binding of φ29 DNA polymerase residue Lys529 with TP and DNA during replication of TP-DNA.

## Supporting Information

Figure S1 **Mutations at φ29 DNA polymerase residue Lys529 impair DNA binding at the polymerisation active site.** The 5′-labelled hybrid molecule 15mer/21mer, depicted on top of the figure, was incubated either with the wild-type or with the indicated mutant derivative, under the conditions described in Materials and Methods. After non-denaturing gel electrophoresis, the mobility of free DNA and that of the polymerase-DNA complex was detected by autoradiography.
(TIF)

Figure S2 **3′-5′ exonuclease activity of mutants K529A and K529E**. The assay was performed as described in Materials and Methods, using ^32^P-labeled 15mer/21mer as dsDNA substrate (A) and ^32^P-labelled 15mer as ssDNA substrate (B). After incubation for the indicated times at 25°C, degradation of the labelled DNA was analysed by electrophoresis in 8 M urea-20% polyacrylamide gels and autoradiography. Total degradation was calculated as indicated in Materials and Methods. The position of 4mer degradation intermediate of the sp1 substrate (15mer) is indicated.
(TIF)

Figure S3 **Hydrolysis of pNP-TMP.** The assay was carried out as described in Materials and Methods. Hydrolysis was studied by monitoring *p*-nitrophenol production that was plotted against time and adjusted to a rectangular hyperbola by least-squares non-linear regression, using Kaleidagraph 3.6.4. software. The DNA polymerase wild-type is represented by full squares, and the mutants K529A and K529E by open squares and open circles, respectively.
(TIF)

Figure S4 **3′-5′ exonucleolytic degradation of short substrates by point mutants in residue Lys529 of φ29 DNA polymerase.** The assay was carried out in the conditions described in Materials and Methods, using as substrate either the 5mer GATCA (A) or the 4mer GATC (B) substrates. After incubation for the indicated times at 25°C, degradation of the labelled DNA was analysed by electrophoresis in 8 M urea-20% polyacrylamide gels and autoradiography. Total degradation was calculated as indicated in Materials and Methods. The position of different degradation intermediates is indicated. Mean activity values relative to the wild-type are shown in Table 1. C: control DNA.
(TIF)

Figure S5 **Interaction of the 5′ nucleotide of the oligo (dT)_5_ with Lys529.** Crystallographic data corresponding to φ29 DNA polymerase with an oligo (dT)_5_ bound at its exonuclease domain are from Protein Data Bank (PDB) ID 1XHZ [12]. Picture shows residue Lys529 (represented as spheres) interacting with the 5′ phosphate of the ssDNA (coloured in grey). Polymerisation and exonuclease domains are represented as green and red ribbons, respectively.
(TIF)
