# Supplementary figures and images for: Dual Role of φ29 DNA Polymerase Lys529 in Stabilisation of the DNA Priming-Terminus and the Terminal Protein-Priming Residue at the Polymerisation Site
Source: PLoS One. 2013 Sep 4;8(9):e72765. doi: 10.1371/journal.pone.0072765 (PMC3762793; doi:10.1371/journal.pone.0072765)

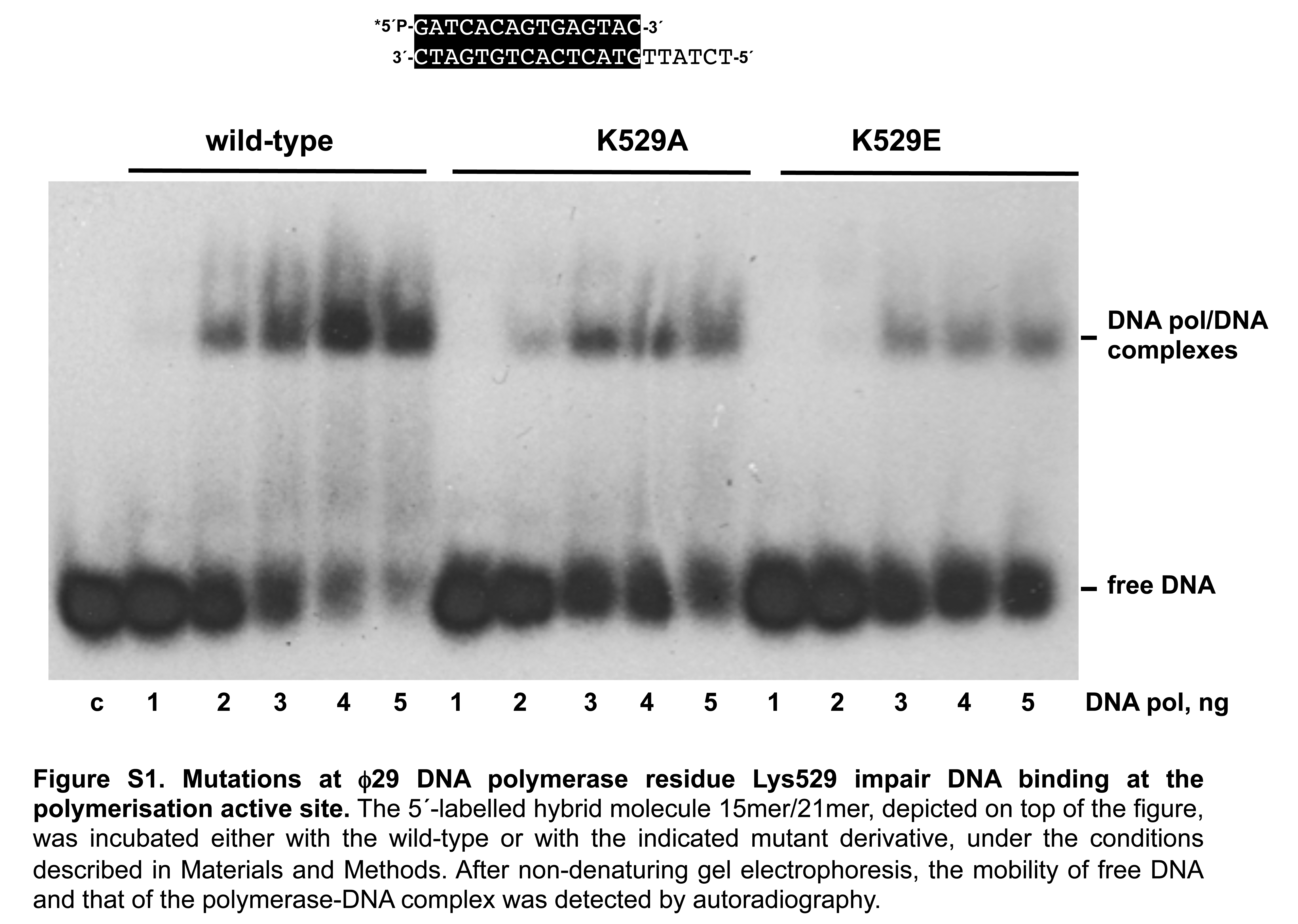

Supplement: Figure S1 — Mutations at φ29 DNA polymerase residue Lys529 impair DNA binding at the polymerisation active site. The 5′-labelled hybrid molecule 15mer/21mer, depicted on top of the figure, was incubated either with the wild-type or with the indicated mutant derivative, under the conditions described in Materials and Methods. After non-denaturing gel electrophoresis, the mobility of free DNA and that of the polymerase-DNA complex was detected by autoradiography. (TIF) [file pone.0072765.s001.tif]

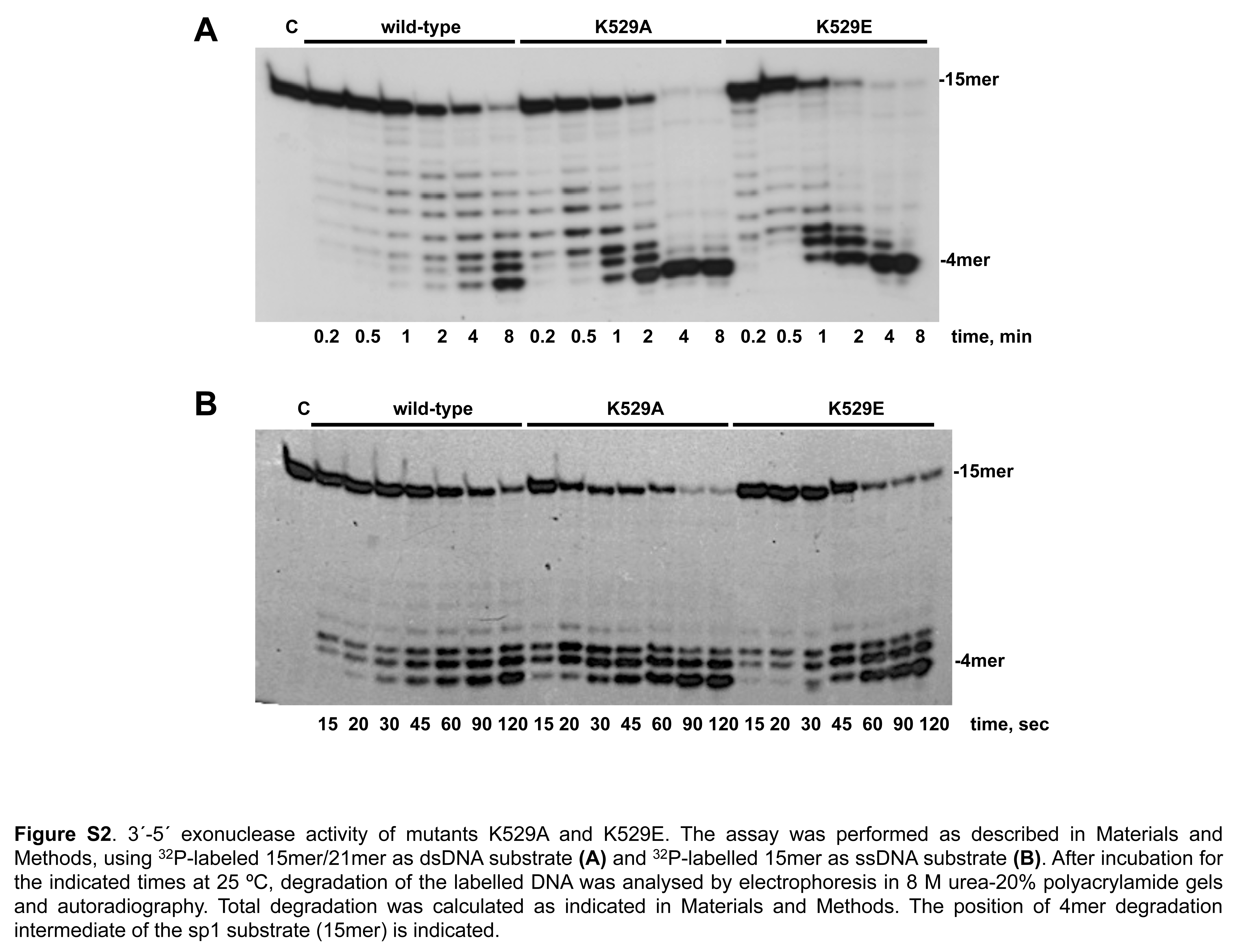

Supplement: Figure S2 — 3′-5′ exonuclease activity of mutants K529A and K529E. The assay was performed as described in Materials and Methods, using 32P-labeled 15mer/21mer as dsDNA substrate (A) and 32P-labelled 15mer as ssDNA substrate (B). After incubation for the indicated times at 25°C, degradation of the labelled DNA was analysed by electrophoresis in 8 M urea-20% polyacrylamide gels and autoradiography. Total degradation was calculated as indicated in Materials and Methods. The position of 4mer degradation intermediate of the sp1 substrate (15mer) is indicated. (TIF) [file pone.0072765.s002.tif]

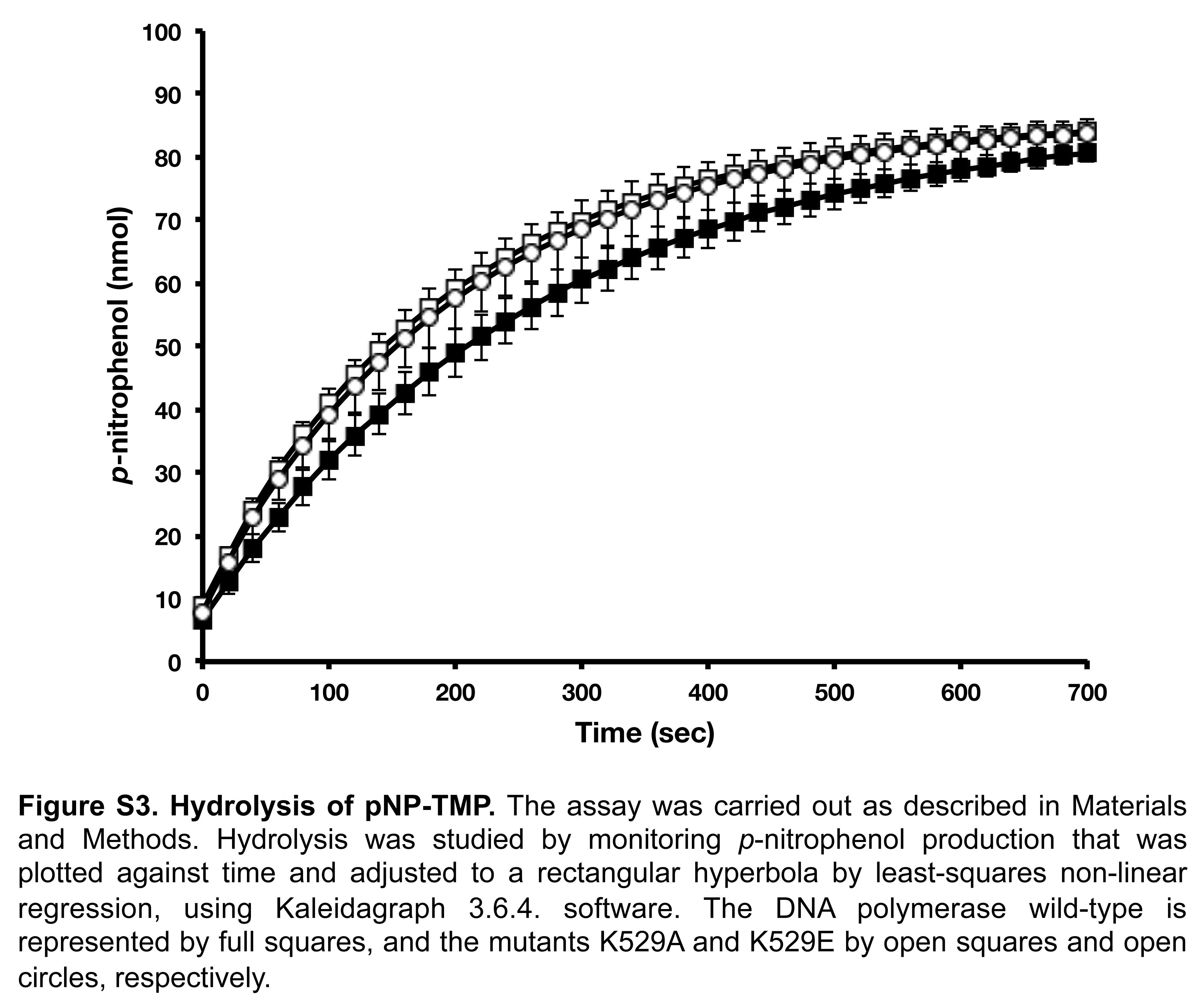

Supplement: Figure S3 — Hydrolysis of pNP-TMP. The assay was carried out as described in Materials and Methods. Hydrolysis was studied by monitoring p-nitrophenol production that was plotted against time and adjusted to a rectangular hyperbola by least-squares non-linear regression, using Kaleidagraph 3.6.4. software. The DNA polymerase wild-type is represented by full squares, and the mutants K529A and K529E by open squares and open circles, respectively. (TIF) [file pone.0072765.s003.tif]

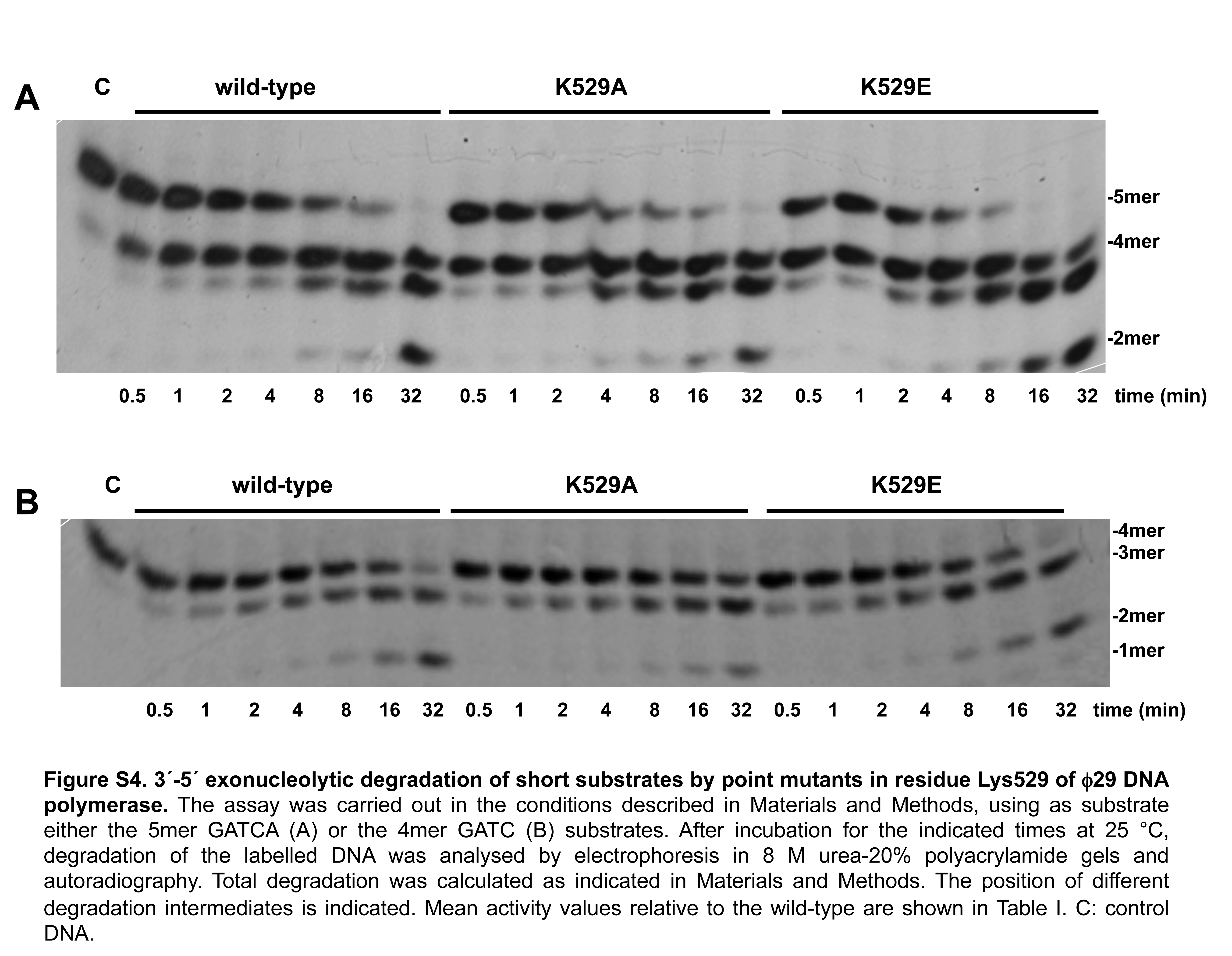

Supplement: Figure S4 — 3′-5′ exonucleolytic degradation of short substrates by point mutants in residue Lys529 of φ29 DNA polymerase. The assay was carried out in the conditions described in Materials and Methods, using as substrate either the 5mer GATCA (A) or the 4mer GATC (B) substrates. After incubation for the indicated times at 25°C, degradation of the labelled DNA was analysed by electrophoresis in 8 M urea-20% polyacrylamide gels and autoradiography. Total degradation was calculated as indicated in Materials and Methods. The position of different degradation intermediates is indicated. Mean activity values relative to the wild-type are shown in Table 1. C: control DNA. (TIF) [file pone.0072765.s004.tif]

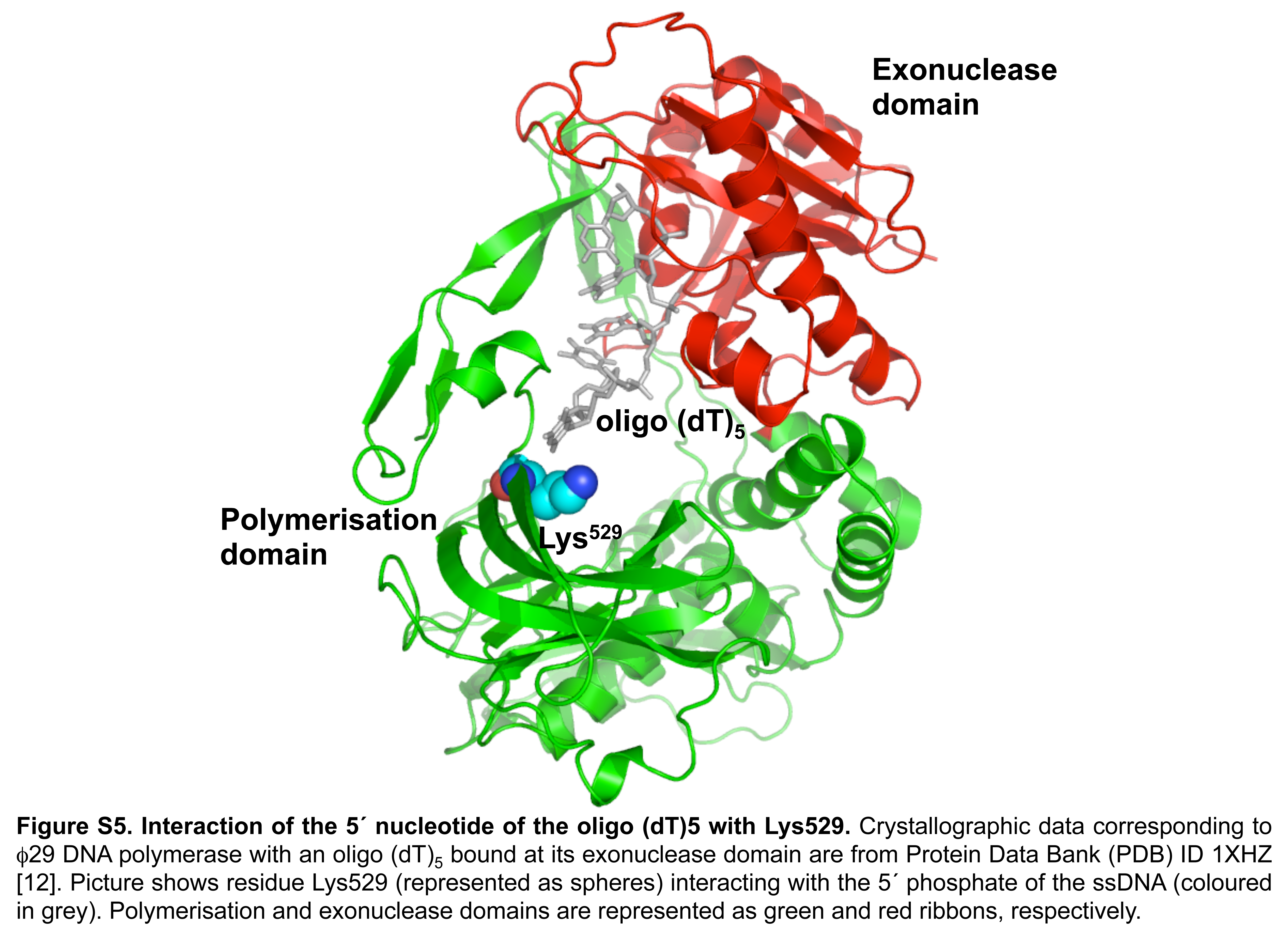

Supplement: Figure S5 — Interaction of the 5′ nucleotide of the oligo (dT)5 with Lys529. Crystallographic data corresponding to φ29 DNA polymerase with an oligo (dT)5 bound at its exonuclease domain are from Protein Data Bank (PDB) ID 1XHZ [12]. Picture shows residue Lys529 (represented as spheres) interacting with the 5′ phosphate of the ssDNA (coloured in grey). Polymerisation and exonuclease domains are represented as green and red ribbons, respectively. (TIF) [file pone.0072765.s005.tif]
